# Exploring long-term protection of normal human fibroblasts and epithelial cells from chemotherapy in cell culture

**DOI:** 10.18632/oncotarget.248

**Published:** 2011-03-28

**Authors:** Pasha Apontes, Olga V. Leontieva, Zoya N. Demidenko, Fengzhi Li, Mikhail V. Blagosklonny

**Affiliations:** Roswell Park Cancer Institute, Elm & Carlton Streets, Buffalo, NY 14263, USA

**Keywords:** cancer, chemotherapy, chemoprotection, normal cells, cyclotherapy, rapamycin, metformin, p53

## Abstract

Killing of proliferating normal cells limits chemotherapy of cancer. Several strategies to selectively protect normal cells were previously suggested. Here we further explored the protection of normal cells from cell cycle-specific chemotherapeutic agents such as mitotic inhibitors (MI). We focused on a long-term cell recovery (rather than on a short-term cell survival) after a 3-day exposure to MI (paclitaxel and nocodazole). In three normal human cell types (RPE, NKE, WI-38t cells) but not in cancer cells with mutant p53, pre-treatment with nutlin-3a, a non-genotoxic inducer of wt p53, caused G1 and/or G2 arrest, thus preventing lethal mitotic arrest caused by MI and allowing normal cells to recover after removal of MI. Rapamycin, an inhibitor of the nutrient-sensing mTOR pathway, potentiated the protective effect of nutlin-3a in normal cells. Also, a combination of rapamycin and metformin, an anti-diabetic drug, induced G1 and G2 arrest selectively in normal cells and thereby protected them from MI. A combination of metformin and rapamycin also protected normal cells in low glucose conditions, whereas in contrast it was cytotoxic for cancer cells. Based on these data and the analysis of the literature, we suggest that a rational combination of metformin and rapamycin can potentiate chemotherapy with mitotic inhibitors against cancer, while protecting normal cells, thus further increasing the therapeutic window.

## INTRODUCTION

Microtubule-targeting agents or mitotic inhibitors are one of the cornerstones of modern chemotherapy [1-5]. Despite different effects on microtubules and tubulin, a variety of structures and binding sites, all microtubule-active drugs at low concentrations kill proliferating (cycling) cells, by causing fatal mitotic arrest [6-9]. In addition to killing cancer cells, these mitotic inhibitors (MI) can kill normal cycling cells (bone marrow cells, hair follicles, mucosal and epithelial cells), thus causing certain side effects. Side effects may not only be devastating, but they also limit anticancer therapy. The goal is to protect proliferating normal cells from the cytotoxicity of MI, without protecting cancer cells.

Given that MI cannot possibly cause mitotic arrest in the cells that do not enter mitosis, a transient G1 and/or G2 arrest (protective arrest) must protect cells from MI. How can we cause a protective G1/G2 arrest selectively in normal cells but not in cancer cells? Whereas all normal cells have wt p53, this tumor suppressor is mutant or lost in 50% of cancers [10]. This absence provides a means for selective protection of normal cells without protecting cancer cells lacking wt p53 [11-13]. Thus, induction of p53 can arrest cells in G1 and G2 phases, preventing their entry into mitosis. Specifically, low concentrations of DNA damaging drugs (DDD) induce wt p53 and arrest cells in G1 and G2, thus protecting cells from mitotic inhibitors [14-17]. In paired cell lines, DDD protected cells with wt p53 but not cells lacking p53 and p21 [14]. However, the effects of DDD are poorly reversible. While mitotic arrest and polyploidization were prevented (a short term protection), the prospect on long-term cell survival and recovery of proliferation remained unclear. Also DDD can protect some cancer cell lines lacking p53 [15]. It was given consideration from the start that less toxic and more selective drugs should be found to induce p53 for the optimal protection of normal cells [14].

Nutlin-3a, an inhibitor of Mdm2, induces wt p53 without causing DNA damage [18, 19]. Also, its effect is strictly p53-dependent [20]. In paired and isogenic cancer cell lines (with and without wt p53), nutlin-3a selectively protected cells with wt p53 [16, 21, 22]. Importantly, nutlin-3a provided a long-term protection from paclitaxel to skin-derived fibroblast cell line in culture, so that cells proliferated after removal of paclitaxel [21]. This outstanding result needed to be further confirmed in other normal cell types including epithelial cells.

Nutlin-3a is an experimental therapeutic and is not approved yet for clinical use. Therefore, we also planned to investigate clinically approved drugs that might protect normal cells from MI. Cancer cells are characterized by dysregulation of the PI-3K/mTOR pathway [23-26]. It was hypothesized that the mTOR inhibitor rapamycin may protect normal cells from cycle-dependent chemotherapy [27]. Although seemingly unrelated, a remarkable study by Longo and co-workers showed that fasting protected mice from the toxicity of chemotherapy [28-30] and, most importantly, abrogated side effects of chemotherapy in patients with cancer [31]. We suggest that protective effect of fasting may in part be due to inhibition of the nutrient-sensing mTOR pathway in normal cells. Fasting decreases blood levels of nutrients (glucose, amino acids), IGF-1 and insulin, which otherwise activate mTOR in the organism [32-37].

Metformin, an anti-diabetic drug, can in part substitute for fasting because it decreases levels of glucose and insulin [38, 39]. Metformin deactivates mTOR in mice [40]. Also, metformin inhibits the mTOR pathway in cell culture [41-43]. Both metformin and rapalogs (rapamycin and its analogs) are clinically approved drugs. Rapalogs are also approved for cancer therapy [44, 45]. Metformin and rapamycin potentiate chemotherapy against cancer [46]. Furthermore, rapalogs and metformin in combination with chemotherapy (including MI) are undergoing clinical trials [47-50].

Here we investigated whether nutlin-3a, rapamycin, metformin and their combinations can protect 3 normal cell types: WI-38t fibroblasts, RPE (retinal pigment epithelial) and NKE (normal kidney epithelial) cells, without protection of MDA-MB-231 breast cancer cells with mutant p53. We investigated whether normal cells were fully protected and recovered upon removal MI. We identified effective combinations of rapamycin with both metformin and nutlin-3a for protection of normal cells from MI.

## RESULTS

### Protection of WI-38t cells by nutlin-3a

**Figure 1:**
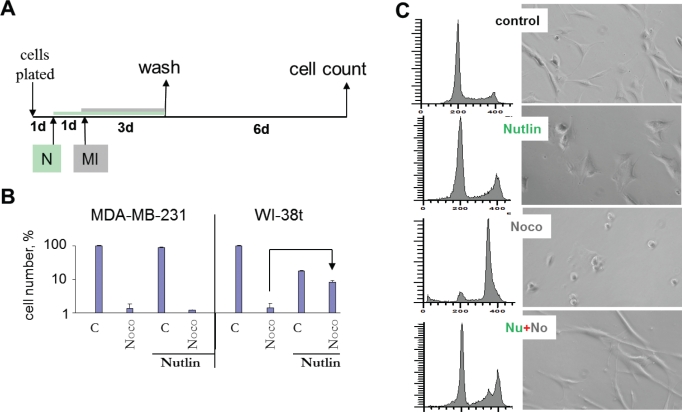
Nutlin-3a protects cells by preventing mitotic arrest caused by nocodazole (A) Drug treatment schedule: MI was added 1 day after nutlin-3a. 3days later all drugs were washed off and cells were allowed to recover for 6 d and then counted. (B) Selective protection of WI-38t, but not MDA-MB-231 cells, from the cytotoxic effect of nocodazole. 10,000 cells were plated per well (in 6 well plates), treated as shown in panel A and counted after 6 days. The results are shown as % of control, in log scale. (C) Prevention of toxic mitotic arrest by non-toxic G1 and G2 arrest. WI-38-t cells were pre-treated with 2.5 μM nutlin-3a, and then were treated with 200 nM nocodazole. After 24 hours, cells were microphotographed (right panel), collected and analyzed by flow cytometry.

To evaluate long-term protection, we determined cell numbers six days after removal of mitotic inhibitors (MI) such as nocodazole and paclitaxel (Fig. 1A). We initially used nocodazole (Noco) because it is easily removable by washing cell culture. Cells were pretreated with nutlin-3 and the next day nocodazole was added. After 3 days of treatment with nocodazole, cells were washed and cultured in the fresh medium for 6 days to allow recovery of the protected cells (Fig. 1 A). Nocodazole alone decreased cell numbers more than 50-fold compared with control in both MDA-MB-231 and WI-38t (Fig. 1B). Pretreatment with nutlin-3a did not protect MDA-MB-231 cells, but completely prevented the effects of nocodazole in WI-38t cells (Fig. 1B). Nutlin-3 caused G1 and G2 arrest (Fig. 1C). Nocodazole arrested cells with 4N DNA content, corresponding to mitotic arrest as evidenced by mitotic/round cells visualized on live microscopy (Fig. 1C). Pretreatment with nutlin-3a (Nu) completely prevented nocodazole-induced mitotic arrest (Fig. 1C, Nu+Noco). Cell cycle distributions of nutlin-pretreated cells were almost identical with (+Noco) and without nocodazole (Fig. 1C). Thus, G1 and G2 arrest caused by nutlin-3a prevented mitotic arrest caused by nocodazole.

**Figure 2:**
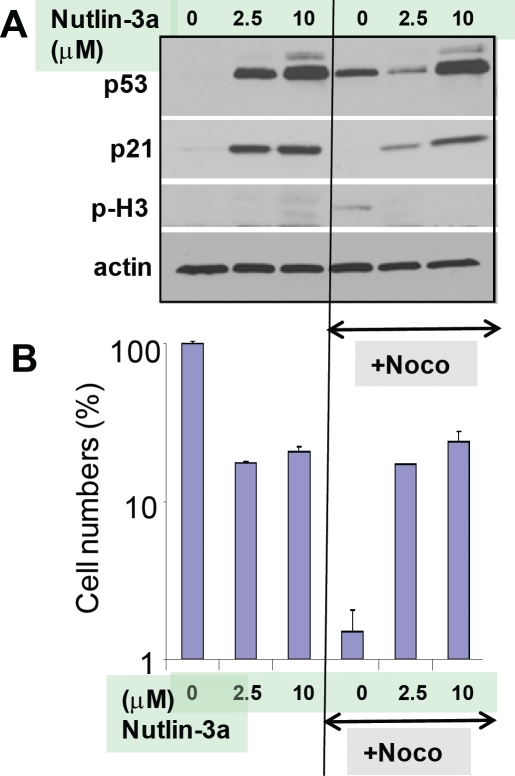
Protective induction of p53/p21 by nutlin-3a in WI-38t cells **A.** Immunoblot. WI-38t cells were pre-treated with indicated concentrations of nutlin-3a (2.5 μM and 10 μM), and then treated with 200 nM nocodazole. The next day, cells were lysed and immunoblot was performed as described in the Methods section. **B.** 50, 000 cells were plated per well (in 6 well plates) and treated as shown in Fig. 1A. The results present cell numbers expressed as % of control in log scale. Note: Cell numbers in Nutlin+Noco exceeded plated cell numbers almost 10-fold, indicating active cell proliferation after drug removal (not shown).

As expected, nutlin-3a induced p53 and p21 (Fig. 2A). Nocodazole alone induced p53 without inducing p21. This is consistent with induction of p53 during prolonged mitotic arrest [7, 51, 52]. Isolated induction of p53 (without induction of p21) by nocodazole is a consequence of mitotic arrest [7, 52]. At both concentrations of nutlin-3a (2.5 μM and 10 μM), cells were completely protected from nocodazole: cell numbers were equal after treatment with nutlin-3a alone and nutlin-3a plus nocodazole (Fig. 2B).

### Protection of RPE cells by nutlin-3a

**Figure 3:**
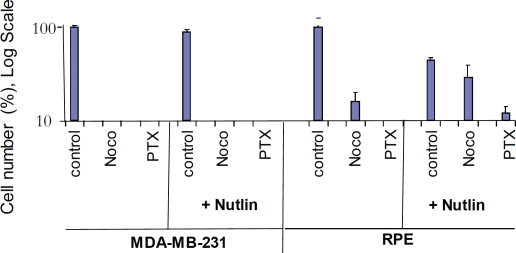
Nutlin-3a protects normal retinal pigment epithelial (RPE-19) cells but not MDA-MB-231 cells 10,000 cells were plated and treated the next day with 2.5 μM nutlin-3a or left untreated. The next day, cells were treated with either 200 nM nocodazole, 50 nM Taxol (paclitaxel) or left untreated. Cells were counted 6 days after wash because control cells reached confluence at that time. The results are shown as % of control, in log scale. Note: Nutlin+Noco, final cell numbers exceeded plated cell numbers, indicating cell proliferation after drug removal.

Nutlin-3a also causes reversible arrest in RPE cells, so that cells could resume proliferation after nutlin-3a was removed [53, 54]. We next investigated whether this arrest can protect RPE cells from the toxicity of MI. Pretreatment with nutlin-3a partially abrogated the toxicity of nocodazole and paclitaxel (Taxol) in RPE cells but not in cancer MDA-MB-231 cells with mutant p53 (Fig. 3). The effect of paclitaxel (PTX) was less reversible than the effect of nocodazole because PTX is poorly washable from the cell culture.

**Figure 4:**
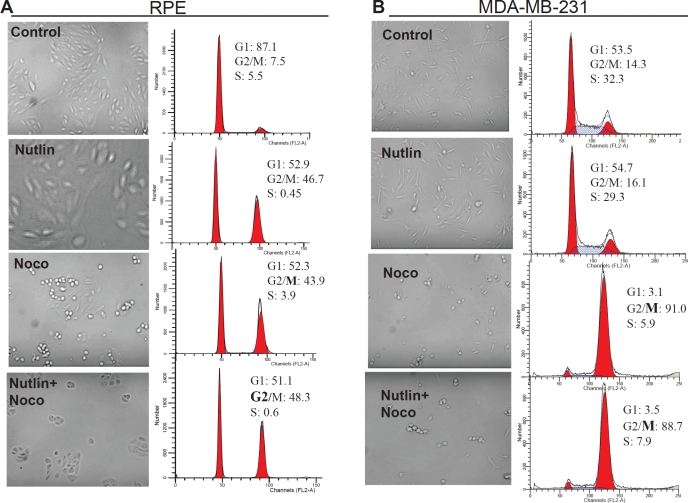
Nutlin-3a prevents toxic mitotic arrest by causing non-toxic G2 arrest in RPE cells RPE (A) and MDA-MB-231 (B) cells were plated in 6-well plates at 50,000/well. Cells were either pretreated with 2.5 μM nutlin-3a or left untreated before addition of 200 nM nocodazole. After 24 hours treatment with nocodazole, cells were microphotographed (left panels), collected and analyzed by flow cytometry (right panels).

Analysis of cell cycle distribution revealed that nutlin-3a caused G2 arrest in RPE cells (Fig. 4). As expected, nocodazole caused mitotic arrest, which was indistinguishable from G2 arrest by flow cytometry, but was evident by the appearance of mitotic cells (Fig. 4A, microphotographs). By arresting cells in G2, nutlin-3a prevented mitotic arrest, thus protecting normal cells. In contrast, nutlin-3a did not cause arrest in MDA-MB-231 cells (Fig. 4B). Therefore, nocodazole caused mitotic arrest in MDA-MB-231 cells both in the presence and the absence of nutlin-3a (Fig. 4B).

### Protection of RPE by drug combinations

**Figure 5:**
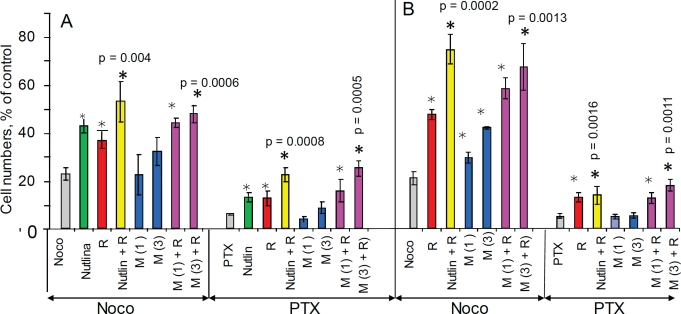
Cytoprotection of RPE cells with nutlin-3a, rapamycin and metformin 10,000 RPE cells were plated in normal (1g/L) glucose medium (panel A) and low (0.5 g/L) glucose medium (panel B) The next day, cells were treated with either 2.5 μM nutlin-3a (Nutlin), 1 and 3 mM metformin (M 1 and M 3), 1 nM rapamycin (R) or left untreated. The next day, cells were treated with either 200 nM nocodazole (Noco) or 50 nM paclitaxel (PTX). 3 days later, the cells were washed and cultured in normal glucose for an additional 10 days and then were counted. The results are shown as % of control (no treatment). Note: in control cells reached confluence during the experiment.

Noteworthy, protection of RPE by nutlin-3a was potentiated by rapamycin, which was slightly cytoprotective by itself (Fig. 5 A). Metformin alone was marginally protective but its effect was also potentiated by rapamycin. A combination of metformin plus rapamycin and a combination of nutlin-3a plus rapamycin afforded comparable protection. Similar results were obtained in low glucose medium (Fig. 5 B).

### Protection of WI-38t by a combination of rapamycin and metformin

**Figure 6:**
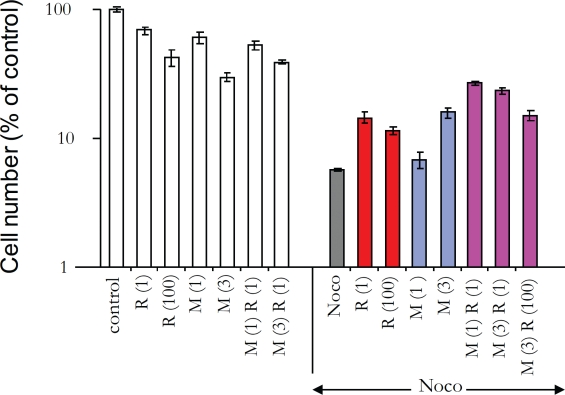
Cytoprotection of WI-38t cells by combining rapamycin and metformin 10,000 WI-38t cells were plated in normal glucose medium The next day, cells were treated with either 1 mM or 3 mM metformin (M 1 and M 3) and 1 nM or 100 nM rapamycin (R 1 and R 100) or left untreated (control). The next day, cells were treated with 200 nM nocodazole (Noco) as indicated. After the 3 days, the cells were washed and cultured for an additional 10 days and then were counted. The results are shown as % of control (no treatment). Note: in control cells reached confluence during the experiment.

**Figure 7:**
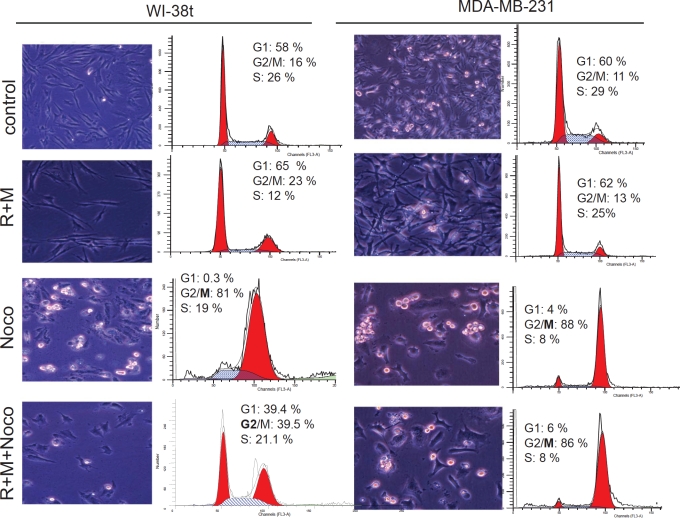
A combination of rapamycin and metformin prevents mitotic arrest in WI-38t cells 50,000 WI-38t and MDA-MB-231 cells were plated in 6-well plates and pre-treated with a combination of 100 nM rapamycin and 3 mM metformin (R+M). The next day, cells were treated with 200 nM nocodazole. After 24 hours, cells were microphotographed (left panels), collected and flow cytometry was performed (right panels).

Next we investigated whether a combination of rapamycin and metformin (R+M) can protect WI-38t cells. This combination (M + R) afforded 2-3 fold protection in WI-38t, but not in MDA-MB-231 cells (Suppl. Fig. 1). Individually, 3 mM metformin and 1 nM-100 nM rapamycin were slightly protective. Combinations of metformin with 1 nM rapamycin were more effective (Fig. 6). We next investigated the mechanism of cytoprotection by rapamycin plus metformin (R+M). The combination (R+M) caused G1/G2 arrest and this partially prevented mitotic arrest caused by nocodazole (Fig. 7). In contrast, R+M did not protect MDA-MB-231 cells (Fig. 7). Noteworthy, the R+M combination was toxic to MDA-MB-231 cells cultured at low glucose (Suppl. Fig. 2).

### Comparison of M+R and nutlin-3a in NKE cells

**Figure 8:**
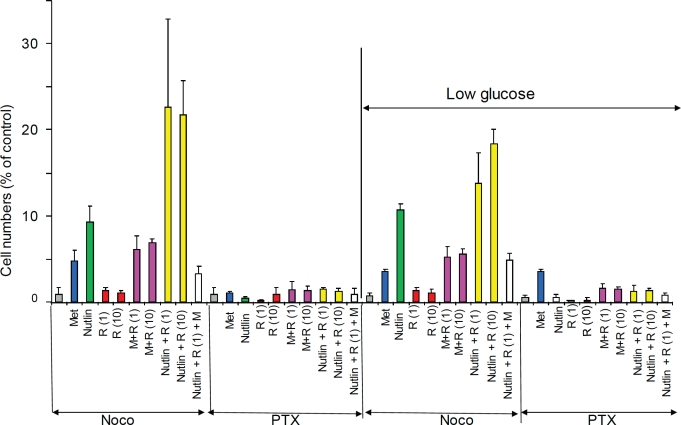
Protection of NKE cells from nocodazole and paclitaxel in normal glucose and low glucose 10,000 NKE cells were plated in either 1g/L glucose or 0.5g/L glucose (low). Cells were pre-treated for 24 hrs with either 2.5 μM nutlin-3a, 1 and 10 nM rapamycin (R1 and R10), 3 mM metformin (M) alone or in combination and then were treated with either 200 nM nocodazole (Noco) or 50 nM Taxol (PTX). After 3 days, cells were washed and cultured for 6 days in fresh medium before counting.

We next tested whether these combinations were protective in a third type of normal cells. Normal kidney epithelial (NKE) cells were partially protected from nocodazole and paclitaxel (PTX) in both normal and low glucose media. Combinations of nutlin-3a with rapamycin (N+R), even though rapamycin alone was not protective, were the most effective in NKE cells (Fig. 8). Combinations of metformin with rapamycin (R+M) were moderately protective (Fig. 8).

## DISCUSSION

The goal of chemotherapeutic cyclotherapy is selective protection of normal cells by exploiting aberrations in cancer cell cycle such as loss of p53 [13, 55-57]. By causing G1 and G2 arrest, nutlin-3a protects cells with wt p53 from mitotic inhibitors. As a proof of principle in paired cell lines, nutlin-3a prevented mitotic arrest, cell death or polyploidization (a short-term protection) [16, 17, 21, 22]. Furthermore, pretreatment with nutlin-3a allowed normal skin-derived fibroblasts to recover from MI treatment, as measured by an increase in cell numbers after 6 days (a long-term protection) in drug-free medium [21]. Here we focused on a long-term protection and extended this observation to WI-38 fibroblasts and two epithelial cell lines: RPE and NKE. As expected, nutlin-3a arrested normal cells in both G1 and/or G2 (depending on cell type) and thus protected them from mitotic arrest caused by MI. While affording a long-term protection to all 3 normal cell types, nutlin-3a did not protect MDA-MB-231 cancer cells in any experiments. Similar results in normal cell lines of different origin indicate that nutlin-3a might also protect relevant normal cells in the organism. Remarkably, Sur *et al* demonstrated that oral administration of Nutlin-3 (200 mg/kg) efficiently protected mice from neutropenia caused by a mitotic inhibitor [20]. This important result indicates that protection in cell culture is indeed applicable to the organism.

We found that rapamycin further potentiated the protective effect of nutlin-3a in three cell lines, most robustly in NKE cells. There are two potential mechanisms. First, rapamycin, as a mild cytostatic agent, may increase the durability of arrest, thus potentiating the protection. Second, to be protective in a long-term, the arrest must be reversible. Nutlin-3-induced arrest is mostly reversible in most cell lines [58, 59]. But nutlin-3a can also induce irreversible senescence, depending on the activity of mTOR, p53 levels and the duration of the arrest [53, 54, 60]. Rapamycin may improve the reversibility of arrest caused by p53 [53]. We are currently investigating the mechanism of potentiation of nutlin-3a by rapamycin (Leontieva et al, MS in preparation).

Since nutlin-3a does not cause arrest in cancer cells lacking p53, the addition of rapamycin cannot possibly potentiate nutlin-3a in cells with mutant p53. Therefore, like nutlin-3a alone, a combination of nutlin-3a plus rapamycin (N+R) does not protect cancer cells with mutant p53. Therefore, the combination of nutlin-3a plus rapamycin can be used for protection of normal cells in patients having tumors with mutant p53. In heterogeneous tumors with co-existing cells having wt and mutant p53, the N+R combination may spare cancer cells with wt p53. This will revert the tumor to a less aggressive phenotype, which is more sensitive to conventional chemotherapy. Therefore, a combination of N+R+MI may be promising in heterogeneous cancers too.

It was shown that fasting, which decreases levels of glucose and insulin and inactivates nutrient-sensing signaling pathways, decreases side-effects of chemotherapy [29]. Like fasting, metformin, an anti-diabetic drug, decreases glucose levels. Also, metformin lowers levels of insulin in women with early breast cancer [38]. Furthermore, metformin decreases tumor growth in mice fed by high-calorie diet [61, 62]. Metformin and rapamycin differently affects nutrient-sensing pathways [63]. Therefore, 2 drugs may in theory potentiate each other. A combination of temsirolimus (an analog of rapamycin) and metformin showed promising results in phase I clinical trial as cancer therapy [43, 64].

Here we demonstrated that a combination of rapamycin and metformin (R+M) caused protective G1 and G2 arrest in normal cells. Furthermore, this combination provided a long-term protection against MI in all three normal cell types. Not only did the R+M combination not protect cancer cells but, in contrast, it was toxic by itself to MDA-MB-231 cells in low glucose conditions. Low glucose levels are toxic to cancer cells, which depend on glucose as substrates for their glycolytic phenotype [65-72]. Noteworthy, metformin decreases glucose levels in the organism.

**Figure 9:**
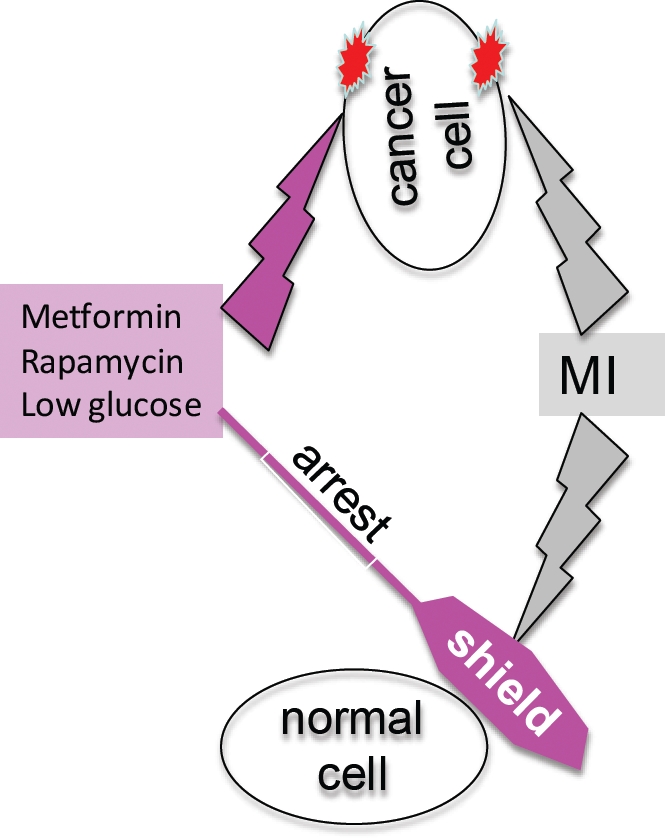
Cyclotherapy: protection of normal cells and unshielding of cancer cells

**Figure 10:**
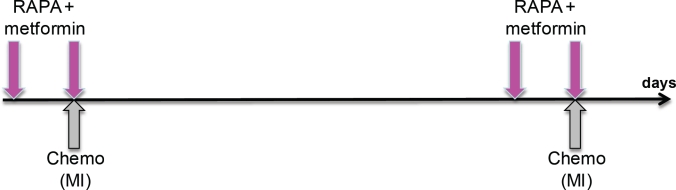
Proposal for potential clinical trials

Metformin enhances susceptibility of p53-/- cells to apoptosis upon nutrient deprivation [73]. In contrast, p53-proficient cells can undergo cell cycle arrest (instead of apoptosis) in low glucose [74]. This explains selective toxicity of metformin to p53-deficient cells in low glucose [73, 74] observed in our study too. Thus a combination of metformin, rapamycin and fasting may be toxic to cancer cells, while protecting normal cells from chemotherapy with MI (Fig. 9), thus increasing therapeutic window. Concentrations of metformin (1 mM) and rapamycin (1 nM) are lower than those achievable in patients [75-80]. We suggest that metformin and rapamycin should be administrated for a 1-2-day period prior and simultaneously with MI such as the Vinca drugs and Taxanes (Fig. 10). In conclusion, based on cell culture data and analysis of the literature, we suggest that nutlin-3a, nutlin-3a plus rapamycin and rapamycin plus metformin may extend the therapeutic window of microtubule-active chemotherapy. Low concentrations of rapamycin and metformin, especially combined with fasting, may be used in the clinic today.

## METHODS AND MATERIALS

### Cell Culture

Cells were cultured in RPMI media purchased from Cellgro (Manassas, VA) supplemented with 5% (v/v) FBS. Rapamycin was purchased from LC Laboratory (Woburn, MA), and metformin, nutlin-3a, nocodazole and paclitaxel were from Sigma-Aldrich (St. Louis, MO, USA). Glucose was added to glucose-free RPMI to desired concentrations or mixed in ratios using RPMI with glucose (2 g/L) and RPMI without glucose. WI-38, WI-38t fibroblasts immortalized by telomerase and retinal pigment epithelial (RPE) ARPE-19 cells were described previously [81], [59]. MDA-MB-231 was from ATCC (Manassas, VA). Normal Kidney Epithelial (NKE) cells were provided by Dr. Katerina V. Gurova (RPCI).

### Flow cytometry

Cells were fixed in 70% ethanol and stained with 25 μg/ml propidium iodide (PI), 0.2% Triton X- 100 and 40 μg/ml RNase in PBS. Flow cytometry was performed using FACScan, Becton Dickinson, San Jose, CA with 10,000-20,000 events per sample. Cell cycle distribution was analyzed using Modfit LT software (Verity Software House Inc., Topsham, ME) with histograms of DNA content profiles on the X-axis and cell numbers on the y-axis.

### Mitotic Index

Following flow cytometry, propidium iodine stained nuclei were visualized on coverslips and mitotic cells were identified by morphological characteristics allowing G2 phase verses mitotic cells to be distinguished. Cells were photographed on a Zeiss Axioplan 2 microscope (Thornwood, NY).

### Cell Counting

Cells were counted on Vicell Series Cell Viability Analyzer (Beckman Coulter, Inc., Brea, CA) using Trypan Blue exclusion method to provide a proportion of live and dead cells.

### Immunoblot analysis

Immunoblot was performed as described previously [53]. The following antibodies were used: mouse anti-p53 (Ab-6) from Oncogene, mouse anti-p21 from BD Biosciences (San Jose, CA); rabbit anti-actin from Sigma-Aldrich (St. Louis, MO); secondary goat anti-rabbit and goat antimouse HRP conjugated antibodies were from Chemicon (Billerica, MA) and Bio-Rad (St. Louis, MO), respectively.

## SUPPLEMENTAL FIGURES


